# Binge drinking and alcohol prices: a systematic review of age-related results from econometric studies, natural experiments and field studies

**DOI:** 10.1186/s13561-014-0040-4

**Published:** 2015-02-12

**Authors:** Jon P Nelson

**Affiliations:** Department of Economics, Pennsylvania State University, University Park, PA, 16802 USA

**Keywords:** Binge drinking, Alcohol prices, Youth, Young adults, Systematic review

## Abstract

**Background:**

Heavy episodic (“binge”) drinking of alcohol has serious public health implications, especially for youth and young adults. Previous summaries and surveys have failed to address in a comprehensive manner the effects of alcohol prices on binge drinking by gender or age group.

**Methods:**

A qualitative systematic review is performed for effects of alcohol prices (or tax surrogates) on binge drinking for three age groups: youth, young adults, and adults. Outcomes examined include binge participation, intensity and frequency. Criteria for data collection and potential sources of bias are discussed, including adequacy of price data. Price-binge relationships are judged using a 95% confidence interval (p ≤ 0.05) for statistical significance.

**Results:**

Fifty-six relevant econometric studies were found, with studies and results distributed equally among three age groups. Also found were five natural experiments for tax reductions and six field studies. Null results or mixed results are found in more than half of the studies. The body of evidence indicates that binge drinkers are not highly-responsive to increased prices. Non-responsiveness holds generally for younger and older drinkers and for male and female binge drinkers alike. A limitation of the current literature is that results are only available for higher-income countries.

**Conclusions:**

Increased alcohol taxes or prices are unlikely to be effective as a means to reduce binge drinking, regardless of gender or age group.

**Electronic supplementary material:**

The online version of this article (doi:10.1186/s13561-014-0040-4) contains supplementary material, which is available to authorized users.

## Introduction

Understanding the determinants of excessive alcohol consumption, especially binge drinking, is important for informed alcohol policy and evaluation [1,2]. High-intensity drinkers who consume several drinks within a short time-period increase risks of serious health, safety and social problems for themselves and others [3,4]. For the United States, binge drinking accounts for more than half of an estimated 80,000 annual deaths and three-quarters of $224 billion in economic costs resulting from excessive alcohol consumption [5,6]. Binge drinking is strongly associated with alcohol-impaired driving, and alcohol-related fatalities are 20% of underage fatal accidents [7,8]. Binge drinking patterns vary importantly by age group and gender [6]: prevalence (28.2%) and intensity (9.3 drinks per episode) are highest among young adults in the US, and then decline with age. However, frequency (5.5 episodes per month) is highest among older adults. Binge prevalence among men (23.2%) is more than twice the rate for women (11.4%), and intensity and frequency also are much higher for men. For persons under 18 years, binge drinking is a special concern since excessive use of alcohol and intoxication by youth are closely associated with similar problems in adult populations [9-11]. Approximately 22% of US high school seniors engaged in binge drinking in 2011. Similar drinking patterns and costs are reported for other countries [3,12]. For example, Anderson [3] reports a binge prevalence of 28% for the European Union (EU), with frequency highest among persons aged 15–24 years. One in six (18%) EU youth report bingeing three or more times in the last month, and one in eight (13%) have been intoxicated more than 20 times in their life. Binge drinking is more common in northern European countries, but high prevalence rates also are reported for some southern countries [3,13]. Overall, binge drinking in the United States is estimated to account for 90% of alcohol consumed by youth and young adults and 50% of alcohol consumed by adults [4]. Alcohol data for European countries suggest that binge-drinking of alcohol is more closely associated with alcohol-related problems compared to average per capita use [14].

During the past several decades, economists have devoted considerable research to alcohol prices as determinates of drinking and drinking patterns, including binge drinking. Econometric (“economic”) studies that incorporate prices (or tax surrogates) fall into three general categories: first, population-level studies for average per capita consumption based on aggregate data that include drinkers and non-drinkers alike, regardless of age, gender, or drinking pattern. A vast majority of studies contained in several recent meta-analyses fall into this category [15-18]. Second, individual-level studies of alcohol use (participation, number of drinks per month) that do not include specific measures of heavy or binge drinking. Third, individual-level studies of binge drinking that incorporate alcohol price or tax variables. These studies provide a stronger evidence base for effective alcohol policies that address abusive and high-intensity drinking. Important alternatives to economic studies are natural experiments (e.g., national tax reductions) and field experiments that rely on special surveys. Most price-binge economic studies reviewed below use individual survey data for the United States, but expanded coverage for other countries is possible by including available natural experiments and field studies.

Despite its importance, no previous summary or survey addresses in a comprehensive manner the effects of alcohol prices on binge drinking by age group or gender. Past summaries cover only a few early studies for youth [19-21] or omit prices and taxes as evidence [22]. More recent surveys cover relatively few economic studies for binge drinking. For example, Wagenaar and colleagues [18] examine only 10 individual-level studies for heavy drinking, while Elder and colleagues [23] cover 10 studies for excessive drinking, including two natural experiments. A review by Patra and colleagues [24] focuses on alcohol-related harms, but binge drinking studies are limited to only three economic studies and several natural experiments. Results by age or gender are not reported in past surveys or apply mostly to early studies. In contrast, the present review examines 56 economic studies for binge drinking divided equally among three age groups, including 34 published prior to 2008. Results by gender also are reported. Five natural experiments and six field studies are reviewed. As discussed below, discrepancies in prior reviews arise in part due to different methods required to search the economics literature on alcohol use. Further, several widely-cited studies attempt to draw a general policy link between alcohol prices and excessive alcohol consumption, but evidence cited is mostly drawn from aggregate econometric studies [25-28]. This is incomplete and potentially misleading, since price and tax elasticity estimates for general populations may not apply equally to binge drinkers and other excessive drinkers [29-31]. A comprehensive survey is required to address effects of prices on prevalence, intensity, and frequency of binge drinking for different age groups. To fill this gap, this paper presents a qualitative systematic review of individual-level studies designed to better understand the potential role of economic incentives for reduction of binge drinking.

## Methods

### Literature search strategy

In order to conduct a systematic review, standardized protocol were employed as set forward in PRISMA (Preferred Reporting Items for Systematic Reviews and Meta-Analyses); see [32,33]. Literature searches were conducted for English-language articles that empirically test relationships between binge drinking and alcohol prices or taxes. Search terms used were: binge*, binge drink*, heavy drink*, intoxication*, and price* or tax*, where * is the truncation indicator to include all forms of the root word (e.g., binge, binger, bingeing). No limitations were placed initially on comparison groups, countries, outcomes, or study designs. However, a general strategy followed in many systematic reviews is to limit initial searches to title/abstract combinations of keywords, such as binge drink* AND price* OR tax*. This strategy does not perform well for research in economics as illustrated by comparisons with earlier reviews or by recent meta-analyses, e.g., Nelson [17] reports 135 studies that were not contained in Wagenaar et al. [18], including 102 published prior to 2008. There are several reasons generally for these discrepancies: first, articles in economic journals usually contain brief abstracts (150-words or less) that disclose relatively little about specifics of statistical models or which emphasize only unique aspects of analyses. Structured abstracts are not used by most economics journals. Second, because market price is a variable in virtually all microeconomic research, most titles and abstracts simply omit this keyword as a non-unique aspect of research methods and results. Only early articles are likely to emphasize price or tax results for binge drinking. Third, many recent econometric articles are not focused on binge drinking per se, but rather on adverse outcomes possibly affected by this and similar drinking patterns, such as schooling, employment, earnings, violence, and drink-driving. Two-stage econometric models estimated in this research do not always report first-stage results for drinking or fail to disclose in the abstract that the paper contains results for alcohol prices. As a consequence, it was necessary to modify conventional search strategies, so initial searches were for, say, binge drink* in the title/abstract and price* OR tax* in the full text. A cost of this approach is that many articles do not include empirical results, making it necessary to manually screen articles by examining text and tables for price/tax estimates and dependent variables for binge drinking. An on-line bibliography containing over 575 studies reflects this modified search process (available from the author upon request).

**Figure 1:**
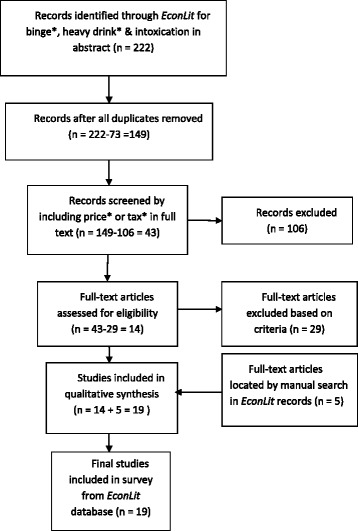
**Flow diagram for EconLit search.**

The main economic database was *EconLit*, which is part of EBSCOhost. For unpublished materials in economics, such as working papers, databases used were Social Science Research Network (SSRN), RePEc Ideas, and Dissertation and Theses portion of ProQuest. Searches focused on economics also were conducted using Google Scholar, JSTOR, ProQuest, and Wiley Online Library. Two public health databases were queried, MEDLINE (PubMed) and EMBASE. Except for natural experiments and field studies, relatively fewer articles (23 out of 72) were found using public health databases since most relevant studies are published in economics journals. Prior reviews and the on-line bibliography were used to trace references compiled in earlier work [17,34]. Figure 1 illustrates search results obtained using *EconLit*, while Table 1 illustrates difficulties encountered if initial searches were limited to keywords in title and abstract. There are 72 entries in Table 1, but only half of the entries would be found by conventional search procedures.

**Table 1:** **Frequency of keywords in title or abstract: primary studies**

**Study; (a) = article; country if not USA**	**Binge drink**	**Heavy drink**	**Intoxication**	**Price**	**Tax**	**No keywords**	**No abstract**
**Youth studies**							
Bhatt (2011)* [35] (a)	x			x			
Carpenter et al. (2007)* [36] (a)		x			x		
Chaloupka & Laixuthai (1997) [37] (a)						x	x
Chatterji (2001) [38]						x	x
Cowan (2011) [39] (a)						x	
Dee (1999b) [40] (a)				x	x		
Dee (1999c) [41] (a)					x		
Dee & Evans (2003) [42] (a)						x	
Grossman (2005) [43]				x	x		
Laixuthai & Chaloupka (1993)* [44] (a)		x		x	x		
Markowitz (2001) [45] (a)						x	x
Medina (2011) [46]*	x			x			
Nair (2001) [47]						x	x
Nelson (2008)* [48] (a)	x				x		
Renna (2007) [49] (a)	x	x					
Saffer & Dave (2006)* [50] (a)	x			x			
Xuan et al. (2013)* [51] (a)	x				x		
**Young adult studies**							
Bray (2000) [52]						x	
Bray (2005) [53] (a)		x					
Chaloupka & Wechsler (1996)* [54] (a)	x			x			
Cook & Moore (1994) [55] (a)						x	x
Cook & Moore (2001) [56]						x	x
Cowell (2006) [57] (a)	x						
French & Maclean (2006) [58] (a)						x	
Gius (2003)* [59] (a)	x				x		
Grossman et al. (1987) [60]						x	x
Keng (1998)* [61]	x	x		x			
Keng & Huffman (2007)* [62] (a)	x			x			
Kenkel (1993) [63] (a)						x	x
Nelson (2008)* [48] (a)	x				x		
Powell et al. (2002) [64]	x						
Rhoads (2010)*	x			x	x		
Sutton & Godfrey (1995)* [65] (a), UK		x		x			
Wechsler et al. (2000)* [66] (a)	x			x			
Weitzman et al. (2003)* [67] (a)	x			x			
Williams et al. (2005)* [68] (a)		x		x			
Wolaver (2007) [69] (a)	x	x					
Wolaver et al. (2007a) [70]	x	x					
Wolaver et al. (2007b) [71]	x	x					
**Adult studies**							
Asgeirsdottir (2012)* [72], Iceland		x		x			
Ayyagari et al. (2013)* [29] (a)		x		x	x		
Blumberg (1992) [73]						x	x
Byrnes et al. (2013) [74] (a), Australia				x	x		
Cook (2007) [75]						x	x
Davalos et al. (2012) [76] (a)	x						
Dee (1999a) [41]					x		
Gius (2002)* (a) [77]	x				x		
Kenkel (1993) [63] (a)						x	x
Kenkel (1996)* [78] (a)		x			x		
Ludbrook et al. (2012)* [79] (a), UK		x			x		
Manning et al. (1995)* [31] (a)		x		x			
McLellan (2011)* [80]	x	x		x	x		
Nelson (2008)* [48] (a)	x				x		
Popovici & French (2013) [81] (a)	x						
Rhoads (2010)*	x			x	x		
Sloan et al. (1995)* [82] (a)		x		x			
Stout et al. (2000)* [83] (a)		x		x			
Terza (2002) [84] (a)					x		
Zhang (2010)* [85] (a)	x				x		
**Experiments & field studies**							
Chung (2013)* [86] (a), Hong Kong	x				x		
Clapp et al. (2003) [87] (a)		x					
Gmel (2008) [88] (a), Switzerland					x		
Gustafsson (2010) [89] (a), Sweden					x		
Heeb (2003)* [90] (a), Switzerland	x			x	x		
Helakorpi (2010)* [91] (a), Finland		x		x			
Jamison & Myers (2008)* [92] (a), UK	x			x			
Kuo (2003) [93] (a), Switzerland				x	x		
O’Mara et al. (2009)* [94] (a)			x	x			
Stockwell et al. (1993) [95] (a), Aus		x	x				
Thombs et al. (2008) [96] (a)		x					
Thombs et al. (2009) [97] (a)		x					
Wagoner et al. (2012) [98] (a)		x					
**72 entries – Total checks**	29	24	2	27	26	15	11

* = 34 studies more likely to be located with conventional keyword combinations for title and abstract. x = keyword located in title or abstract. Complete references are in Additional file 2 and more detailed results are in Additonal file 1 for the on-line supplemental tables. Unpublished studies are: S-H Keng. The demand for health, alcohol abuse, and labor market outcomes: a longitudinal study, unpublished Ph.D. dissertation. Ames, IA: Iowa State University; 1998. JK Rhoads. Consequences of tobacco control policies: intended and unintended, unpublished Ph.D. dissertation. Chicago: University of Illinois at Chicago; 2010.

### Identification of primary studies and quality criteria

Identification of primary studies for review was based on the following quality criteria: (1) examines the relationship between alcohol prices (or tax surrogates) and binge drinking or other measures of heavy drinking that can be easily interpreted as binge drinking (e.g., 35+ units of alcohol consumed per week); (2) reports empirical results for a multivariate relationship, including price/tax regression estimates and standard errors (t-statistics or p-values); (3) reports sufficient information about measures of alcohol consumption, measures of alcohol prices or taxes, other control variables, and average age(s) of survey respondents; and (4) contains empirical results for binge participation, intensity, or frequency. In two cases, correspondence with authors obtained required information. Most econometric studies use individual-level survey data, but two included studies use survey data aggregated to the state level and two studies use aggregate national US data. Natural experiments are based on country-level tax reductions and individual-level surveys, but these studies do not directly incorporate price or tax variables. Field studies are based on random and self-selected interviews with college-aged respondents, with self-reported or observed prices and pricing methods. Studies were excluded if the following criteria were met: (1) based on a laboratory experiment; (2) reports only simple correlations; (3) regression estimates for prices/taxes or standard errors are not reported; (4) uses interrupted time-series analysis; and (5) study is an undergraduate research paper. No studies were excluded for bias reasons, but in several cases there are potential biases that require comment. Many primary studies include results for other drinking behaviors, but only binge drinking results are examined in this review. Most exclusions occur because studies simply do not include or do not report alcohol prices/taxes as a determinant of binge drinking.

### Data collection

Data collected from each study include sample population, subpopulations (age, gender, race), survey employed, average age or age range of respondents, measure(s) of binge drinking as outcomes, measure(s) of alcohol prices or taxes as interventions, statistical method(s) employed, control variables included in the model (e.g., income, demographics), and robustness tests. Complete data in narrative form are in Additional file 1, and complete references are in Additional file 2. The basic result in each study is the level of statistical significance of a price/tax coefficient for a given age group or gender. The summary measure in this review is statistical significance for a price or tax coefficient at the 95% confidence level or better (p-value ≤ 0.05). Results are analyzed according to estimated average age of respondents in each study or sample: youth (ages < 18 yrs.); young adults (ages 18–26 yrs.); and adults (ages > 26 yrs.).

Quantitative coefficient estimates for a meta-analysis were not collected due to diversity of models and results, e.g., participation and frequency elasticities are not comparable, and price and tax elasticities are not comparable [17]. Small samples sizes also are an issue for a meta-analysis. For example, there are only five studies of binge participation by young adults. Meta-analyses of quantitative estimates should correct for publication bias and study heterogeneity [17], which is difficult with small samples. In most economic studies, intuition and theory lead to a qualitative prediction about a parameter, with parameter magnitude based on empirical methods. Thus, a systematic review tests the robustness of qualitative predictions. While qualitative reviews have limitations due to “vote-counting” bias, they allow structured summaries of an evidence base that are useful to future researchers and policymakers.

### Price data limitations

Price data used in primary studies are not obtained from survey respondents and must be imputed based on respondents’ place of residence (state or city). For the US, most researchers have used one of two approaches to measurement: (1) alcohol prices from surveys conducted by American Chamber of Commerce Researchers Association (ACCRA); and (2) state alcohol excise taxes as price surrogates. First, alcohol prices are included in ACCRA’s Cost of Living Index (see http://www.coli.org/), published quarterly for 300 medium and large US cities. Shelf prices are reported for one brand each of beer, wine, and blended whiskey. However, ACCRA data do not capture the full spectrum of alcohol prices [99,100], and geographic details are limited. Young and Bielinska-Kwapisz [101] examine measurement errors and endogeneity of ACCRA prices for demand for alcohol for a panel of 49 states in 1982–1997. Depending on model specification and econometric method, they find substantial variation of price elasticity estimates, which they conclude is evidence of measurement error. Ruhm and colleagues [102] compare ACCRA prices to prices from Universal Product Code (UPC) scanner data on grocery store alcohol sales. They show that in most markets ACCRA prices are higher for beer and spirits and lower for wine. Using National Epidemiological Survey data, they demonstrate that ACCRA data fail to yield stable estimates of beer price elasticities.

Second, a widely adopted alternative is to use state alcohol excise taxes, especially beer taxes, as a proxy for beverage prices. (Beer accounts for two-thirds of all alcohol consumed by binge drinkers [103,104].) A prime attraction is that taxes are policy variables. However, state taxes are a small percent of alcohol prices and tax rates have changed infrequently over time. Hence, cross-sectional variation in unobserved prices is likely dominated by non-tax factors and any temporal variation in real tax rates is largely due to general inflation [40]. Young and Bielinska-Kwapisz [105] report that alcohol taxes are poor predictors of beverage prices, especially for beer. Ruhm and colleagues [102] report that beer taxes are poor predictors of alcohol consumption compared to UPC scanner data. As a result of these measurement errors, many tax and ACCRA price coefficients for binge drinking are likely to be biased toward zero.

A related problem is identification of a causal link between state alcohol taxes and drinking outcomes, including excessive drinking and alcohol-related harms. Dee [41] argues that studies reporting a significant tax-binge relationship are plausibly explained by omitted cross-state attributes and unobserved heterogeneity. For example, state-level “drinking sentiment” will tend to be negatively correlated with observed alcohol tax variables. As a result, cross-state variation in taxes may not provide a valid “natural experiment” or may overstate potential impacts of higher taxes as an alcohol policy. Dee argues that models that limit the number of state-specific variables lack a credible identification strategy, which imparts omitted variable bias to estimates of policy responsiveness for taxes. A statistical solution suggested by Dee [41] is to include state fixed-effects in a panel data model (i.e., a binary variable for each state or local area), which captures relatively stable, but unobserved, cross-state differences potentially affecting drinking patterns and behaviors. In Additional file 1, studies are highlighted that include state fixed-effects and other robustness tests.

## Results

### Primary studies

Dropping some duplicate studies, there are 56 econometric results in the database, divided equally among three age groups. Only three non-US economic studies were obtained for Australia, Iceland, and the United Kingdom. There are five natural experiments and six field studies for Australia (1 study), Finland (1), Hong Kong (1), Sweden (1), Switzerland (2), United Kingdom (1), and United States (4). As noted in Table 1, several studies report results for more than one age group. Both published and unpublished materials are included: peer-reviewed articles, 51; book chapters, 6; dissertations, 6; and working papers, 3. There are 32 peer-reviewed articles published in 21-different economics journals, including 10 articles published in three health economics journals. Nineteen articles were published in public health journals, including 13 for natural experiments and field studies. Thirty-five out of 67 studies use data where the end date is 1999 or more recent. Results by age group are summarized in Table 2. Complete references are in Additional file 2 and more extensive results are in Additional file 1, including data sources, average ages, statistical methods, robustness tests, and control variables.

**Table 2:** **Summary of binge drinking and price/tax studies**

**Study & year; H if Harvard CAS sample**	**Binge drinking measures & quantity**	**Price/tax measures**	**Results for statistical significance**
**Youth studies**			
Bhatt (2011) [35]	2+ episodes, 5+ drinks	ACCRA beer	**Significant**
Carpenter et al. (2007) [36]	Any, 5+ drinks	beer tax	Not significant
Chaloupka & Laixuthai (1997) [37]	Any, 5+ drinks	ACCRA beer	Signif. 1989, not signif. pooled
Chatterji (2001) [38]	Any, 5+ drink; No. episodes	beer tax	Not significant
Cowan (2011) [39]	No. episodes, 5+ drinks	beer tax	Not significant
Dee (1999b) [40]	Any, 5+ drinks	beer tax	Not significant w/ fixed-effect
Dee (1999c) [41]	Any, 5+ drinks	beer tax	Not significant w/ fixed-effect
Dee & Evans (2003) [42]	Any, 5+ drinks	beer tax	Not significant w/ fixed effect
Grossman (2005) [43]	Prevalence, 5+ drinks	BLS beer index	**Significant**
Laixuthai & Chaloupka (1993) [44]	Any, 5+ drinks	beer tax	Signif. 1982, not signif. 1989
Markowitz (2001) [45]	No. episodes, 5+ drinks	beer tax	**Significant**
Medina (2011) [46]	Prevalence, 5+ drinks	BLS beer index	Signif., except males
Nair (2001) [47]	Any, 5+ drinks	beer tax	Signif. males, not females
Nelson (2008) [48]	Prevalence, 5+ drinks	beer tax	Not significant w/ fixed effect
Renna (2007) [49]	2+ episodes, 6+ drinks	beer tax	Not significant
Saffer & Dave (2006) [50]	Any, 5+ drinks	ACCRA ave.	MTF, signif. female, not male
Saffer & Dave (2006) [50]	Any, 5+ drinks	ACCRA ave.	NLSY, not significant
Xuan et al. (2013) [51]	Any, 5+ drinks	beer tax	Not signif. w/ adult binge incl.
**Young adult studies**			
Bray (2000, 2005) [52,53]	3+ episodes, 6+ drinks	beer tax	Men only, not significant
Chaloupka &Wechsler(1996) [54], H	Any, 5/4+ drinks	ACCRA beer	Not significant, both genders
Cook & Moore (1994) [55]	4+ episodes, 6+ drinks	beer tax	Signif. female; not signif. male
Cook & Moore (2001) [56]	4+ episodes, 6+ drinks	beer tax	Not significant, both genders
Cowell (2006) [57]	Any, 6+ drinks; 4+ episodes	beer tax	Men only, not significant
French & Maclean (2006) [58]	No. days intoxicated	beer tax	Signif. male; not signif. female
Gius (2003) [59]	Any, 6+ drinks	Wt. ave. tax	Not significant
Grossman et al. (1987) [60]	No. drinks per day, incl. 6+	BLS prices	Not significant
Keng & Huffman (2007) [62] & Keng (1998) [61],** 2 studies**	4+ episodes, 6+ drinks	ACCRA ave., ACCRA beer	Significant ave. price; not significant for beer price
Kenkel (1993) [63]	No. episodes, 5+ drinks	ACCRA ave.	Signif. female; not signif. male
Nelson (2008) [48]	Prevalence, 5+ drinks	beer tax	Not significant
Powell et al. (2002) [64], H	Any, 5/4+ drink; 3+ episode	Ave price, fix fee	**Signif. price;** fix fee mixed
Rhoads (2010)	Any, 5+ drink; No. episodes	ACCRA ave.	Not significant
Sutton & Godfrey (1995) [65]	Units per week, incl. 36+	Price index	**Men only, significant**
Wechsler et al. (2000) [66], H	Any, 5/4+ drinks	Ave. price, free	**Signif. price;** not signif. free
Weitzman et al. (2003) [67], H	Any, 5/4+ drinks	Ave price, fix fee	**Significant both prices**
Williams et al. (2005) [68], H	Any, 5/4+ drink; No. drunk	Ave price, fix fee	**Signif. price;** not signif. fix fee
Wolaver (2007) [69], H	Any, 5/4+ drink; Any drunk	Ave, fix fee, free	Not significant, both genders
Wolaver et al. (2007a) [70], H	Any, 5/4+ drink; 2+ episode	Ave price, fix fee	Not signif. w/ binge rate incl.
**Adult studies**			
Asgeirsdottir et al. (2012) [72]	Any, 5+ drinks	Price index	Not significant
Ayyagari et al. (2013) [29]	No. episodes, 4+ drink	ACCRA ave.	Not significant
Byrnes et al. (2013) [74]	No. drinks per day, incl. 5+	Price index	Not significant
Cook (2007) [75]	Any, 5/4+ drinks	Wt. ave. tax	**Significant, both genders**
Davalos et al. (2012) [76]	Any, 5/4+ drink; No episode	beer tax	**Significant**
Dee (1999a) [41]	Any, 5+ drinks	Three taxes	Not significant, both genders
Gius (2002) [77]	Any, 6+ drinks	Three taxes	Not significant
Kenkel (1993) [63]	No. episodes, 5+ drinks	ACCRA ave.	**Significant, both genders**
Kenkel (1996) [78]	No. episodes, 5+ drinks	ACCRA ave.	Not signif. except well-info
Ludbrook et al. (2012) [79]	50/35+ units per week	Low price index	**Significant**
Manning et al. (1995) [31]	Any, 5+ drink; No. episodes	ACCRA ave.	Mixed results; signif. part.
McLellan (2011) [80]	Any, 5+ drinks	ACCRA beer	Not significant, w/ fixed effect
Nelson (2008) [48]	Prevalence, 5+ drinks	beer tax	Not significant
Popovici & French (2013) [81]	No. episodes, 5/4+ drinks	ACCRA prices	Not significant, both genders
Rhoads (2010)	Any, 5+ drink; No. episodes	ACCRA ave.	Mixed results; signif. freq.
Sloan et al. (1995) [82]	Any, 5+ drink; No. episodes	ACCRA ave.	Mixed results; signif. freq.
Stout et al. (2000) [83]	Any, 5+ drinks	ACCRA ave.	Not significant
Terza (2002) [84]	Top 10% of use	beer tax	Not significant
Zhang (2010) [85]	Any, 5+ drinks	Three taxes	**Women, significant**
**Experiments & field studies**			
Chung et al. (2013) [86]	Any, 5/4+ drinks	100% tax cut	Not significant
Clapp et al. (2003) [87]	Any, 5+ drinks	Free drinks	Not significant
Gmel et al. (2008) [88]	40/20 g + per day	30-50% tax cut	Not significant, long-run
Gustafsson (2010) [89]	No. units, top 10% of use	45% tax cut	Not significant
Heeb et al. (2003) [90]	Any, 6/4+ drinks	9-50% tax cut	Not significant
Helakorpi et al. (2010) [91]	Any, 6+ drinks	33% tax cut	Mixed results, both genders
Jamison & Myers (2008) [92]	Any, 5/4+ drinks	Price specials	Not significant
O’Mara et al. (2009) [94]	Breath test; no. grams	Price per gram	**Significant** on-premise
Stockwell et al. (1993) [95]	Any, 6/4+ drinks	Price specials	Not significant
Thombs et al. (2008, 2009) [96,97]	Breath test	Price specials	**Fixed fee signif.,** others not
Wagoner et al. (2012) [98]	Any, 5/4+ drinks	Free drinks	**Significant**

Price-binge relationships are judged using a 95% confidence interval (p ≤ 0.05) for statistical significant. Studies with more definitive statistical results are indicated in bold type. Complete references are in Additional file 2 and more detailed results are in Additional file 1 for the on-line supplemental tables. Unpublished studies are: S-H Keng: The demand for health, alcohol abuse, and labor market outcomes: a longitudinal study, unpublished Ph.D. dissertation. Ames, IA: Iowa State University; 1998. JK Rhoads: Consequences of tobacco control policies: intended and unintended, unpublished Ph.D. dissertation. Chicago: University of Illinois at Chicago; 2010.

### Binge drinking definitions

Most economic studies (44 out of 56 table entries) are based on data sources that adopt a standard definition for binge drinking: 5 drinks or more on one occasion (5+ drinks) or 5+ drinks for men and 4+ for women (5/4+ drinks). Eight studies use 6+ drinks based on data in the National Longitudinal Survey of Youth (NLSY). Four studies use varied definitions, including 50/35+ units in a week; top 10% of alcohol use in sample; number of days intoxicated; and number of drunken events. Natural experiments generally use standard binge definitions, while field studies employ measures of drunkenness. While definitions of binge drinking are fairly uniform, measures of drinking behavior differ. For youth studies, 13 economic studies use binge participation (e.g., any binge drinking in past two weeks as a binary outcome); four studies use binge frequency defined as two (three) or more binges in past 14 (30) days or a count of the number of binge episodes; and one study reports results for participation and frequency. For young adults, five studies use participation, six use frequency, two use binge intensity (number of drinks), and six report two measures (e.g., any binge drinking and number of binges in past 30 days). For adults, eight studies use participation, four use frequency, three use intensity, and four use two measures. Given these small samples and diverse measures, emphasis is placed here on the overall results by age category and gender. Lastly, natural experiments use binge participation, while field studies use drinking intensity measures, including breath tests for intoxication.

### Binge drinking results for youth

There are 18 studies or samples for binge drinking by youth, but several are similar in design. Only three of 18 studies – indicated in bold type – report protective results for price/tax interventions, indicating that higher alcohol prices or taxes have a statistically-significant negative effect on youth bingeing (p ≤ 0.05). Ten studies report insignificant or null results for prices or taxes, including NLSY results in Saffer and Dave [50]. The remaining studies report mixed results based on Monitoring the Future (MTF) samples or racial and gender subsamples. Price variables in eight supportive- and mixed-result studies are varied: three use beer taxes; three use ACCRA prices; and two use aggregate Bureau of Labor Statistics (BLS) price indexes. Nine of ten null studies employ state-fixed effects or state-level variables as controls, including two US studies with a control variable for drinking environment (“wetness”) as a determinant of youth binge drinking.

### Binge drinking results for young adults

There are 19 table entries for binge drinking by young adults that can be divided into three groups: (1) five that do not report separate results by gender; (2) seven with results for males or both genders; and (3) seven based on the Harvard College Alcohol Survey (CAS), which uses self-reported information for alcohol prices, price discounts, and price promotions that reduce marginal costs to zero (fixed fees, free drinks). In the first group, Keng and Huffman [61,62] report mixed results that depend on price data used, but four other studies report insignificant relationships between prices/taxes and binge participation or frequency. Also, Cook and Moore [56] report insignificant results for pooled samples of men and women. In the second group, five of seven studies report insignificant results for males, and two of four report insignificant results for females. A United Kingdom study reports a significant negative result for price and male bingeing, but it uses a national price index that might pick-up other data trends. Several US studies include a variety of state-level variables including legal drinking age, drink-driving laws, alcohol availability, and state drinking environment.

One Harvard CAS study by Chaloupka and Wechsler [54] uses ACCRA beer prices at the city level, with insignificant results. Significant negative effects for average price or fixed-fees are reported in three studies; mixed results in one study; and insignificant results in two studies. Two studies, Chaloupka and Wechsler [54] and Wolaver [70], report insignificant price effects for male and female binge drinkers, regardless of legal age. Control variables in CAS studies include demographics, fraternity/sorority (“Greek”) status, peers’ drinking, parents’ drinking, parents’ education, religiosity, alcohol availability, college-level bingeing, and drink-driving laws. This is a diverse set of controls, but half of the studies report mixed or null results.

### Binge drinking results for adults

There are 19 table entries for binge drinking by adults: five studies report that higher prices/taxes reduce binge participation or frequency by adults, but 10 report insignificant or contradictory results. Four studies report mixed results: Kenkel [78] finds a significant effect of price for better-informed drinkers only; Sloan and colleagues [82] find a significant price effect for binge frequency, but not for participation; and Manning and colleagues [31] report that price is significant for binge participation, but not for frequency. Significant price effects are reported for both men and women in studies by Cook [75] and Kenkel [63], but Kenkel [78] also reports insignificant results. In some cases, significant tax elasticities appear to be too large to be credible (e.g., Zhang [85]). Price measures in adult studies include beer taxes; weighted average or multiple taxes; ACCRA beer prices; weighted price or multiple prices; and price indexes. This is a diverse set of price/tax measures, with no apparent impact on pattern of findings.

### Binge drinking results from natural experiments and field studies

Table 2 summarizes results for five natural experiments and six field studies. Natural experiments examine tax reductions on beer and wine (Hong Kong), spirits (Sweden, Switzerland), and all beverages (Finland). Tax reductions range from 100% in Hong Kong to about 30-50% in Nordic countries. A study for Finland by Helakorpi and colleagues [91] finds mixed effects on binge drinking, while four other studies report null effects on binge drinking and heavy drinking more generally. In contrast to economic studies, natural experiments contain fewer control variables and do not directly account for price or tax levels.

There are four field studies for the United States, one for Australia, and one for the United Kingdom. Varied price measures include: free alcohol at events; price discounting such as pitcher specials, drinking game discounts, and buying rounds; fixed-fee/cover charges for all-you-can drink; and average price comparisons by drinking level. A study by Clapp and colleagues [87] reports null results for free alcohol, but Wagoner and colleagues [98] find that free drinks increase binge drinking by both genders. Thombs and colleagues [96] report that fixed-fees increase chances of intoxication among college students, but other price promotions are not significant. Stockwell and colleagues [95] report null results for price discounting among young adults in Australia, while Jamison and Myers [92] and O’Mara and colleagues [94] report mixed results for binge drinking and intoxication. In summary, this is a mixed set of results for pricing methods obtained from field studies

## Review

Overall, null results or mixed results are found in more than half of the studies. For econometric studies, 56 studies contain 30 null results, 12 mixed results, and only 14 studies where a negative relationship with prices is more strongly supported. Findings also are null in more than half of results by age group or by gender. For example, half of the studies report insignificant results for women. Hence, evidence from econometric studies does not strongly support a protective effect for higher alcohol price or tax interventions on binge drinking outcomes, regardless of drinker’s age or gender. Similar results are obtained for natural experiments: four of five studies find no effect of substantial alcohol tax reductions. Field studies report more mixed results as various price measures have been examined, such as price discounting, fixed fees, and free drinks. For example, Stockwell and colleagues [95] p. 1524 conclude that “respondents’ reports as to whether the price of drinks was discounted . . . did not significantly predict either heavy drinking or harm.” Free alcohol is unimportant in four field studies. On the other hand, one field and three CAS studies report that fixed-fee offers are significant in some circumstances. Available evidence for price specials and similar methods is presently mixed and inconclusive. Additional research is required to establish which pricing methods are important for binge drinking, especially for young adults and college students.

In economic studies, two potential sources of bias are: (1) measurement errors in price variables; and (2) omitted variable bias from unobserved state-level attributes that are correlated with state alcohol prices or taxes. As discussed above, price data must be imputed and measurement errors in these data tend to bias estimated coefficients towards zero. A key issue for future research is improved data on prices, where some research efforts have been reported [29,102]. Harvard CAS and field studies also use a variety of self-reported pricing data, yet fail to conclusively support a price-binge relationship. However, omitted variable bias tends to have the opposite effect, with negative coefficients resulting when this bias offsets any measurement errors. (I am grateful to a referee for stressing this point.) Whether or not state-level fixed effects are sufficient to overcome this problem is difficult to assess because few researchers have recognized the problem or made efforts to address the issue. Thus, another key issue for future research is robustness tests that address omitted variable bias, with state fixed-effects high on the agenda. The existing evidence-base therefore has limitations due to these potentially offsetting biases. The evidence does not strongly support an effect of prices on binge drinking, but this reflects measurement and specification errors. Failing to reject the null hypothesis of no relationship does not prove that such a relationship does not exist.

Several other shortcomings of primary studies that underlie this review should be kept in mind. First, most evidence on prices pertains to the United States and a few other higher-income countries. However, natural experiments and field studies for other countries also fail to support alcohol tax increases. Second, more attention might be given to subsamples by age, gender, race, ethnicity, etc. Third, use of similar measures of drinking outcomes combined with subsamples would in the future permit a quantitative synthesis of the binge drinking literature.

## Conclusions

This paper presents a comprehensive review of empirical studies of the relationship between alcohol prices (or tax surrogates) and binge drinking. Results include 22 studies published since 2008, which updates substantially the available evidence-base compared to earlier summaries and reviews. The review includes for the first time, a summary of results for youth, young adults, and adults. A variety of survey-based data are employed in econometric studies, while special surveys and interviews are used for natural experiments and field studies. Binge drinking outcomes include participation, intensity, and frequency. Alcohol price and tax measures include quarterly survey prices, state excise taxes, weighted averages of prices or taxes, price indexes, self-reported prices, and price discounts.

Numerous alcohol policy analyses discuss alcohol tax and price increases as a “best buy” policy for control of excessive or abusive drinking and alcohol-related harms, including binge drinking [25,26,106-110]. For example, Babor and colleagues [26] p. 242 state that “of all the policy options, alcohol taxes is rated as one of the strongest . . . [and] heavier drinkers appear to be as responsive as lighter drinkers, and these policies are effective for younger drinkers as well as adults.” These and similar statements tend to be based on limited literature reviews, older studies, or econometric studies that focus on population-level demand, and not alcohol demands by individual binge drinkers and other excessive drinkers. Although the “law of demand” holds that price and consumption are inversely related, the potential magnitude of the relationship by drinking pattern is an empirical issue. As demonstrated here, a large body of evidence indicates that binge drinkers are not highly-responsive to increased prices. Non-responsiveness holds generally for younger and older drinkers and for male and female binge drinkers alike.

## Additional files

Additional file 1:
**Table S1.** Youth binge drinking studies (ages < 18 yrs.); **Table S2.** Young adult binge drinking studies (ages 18–26 yrs.); **Table S3.** Adult binge drinking studies (ages > 26 yrs.); **Table S4.** Natural experiments and field interview studies for binge drinking.
Additional file 2:
**Primary study references.**

## Abbreviations

ACCRA: American chamber of commerce researchers association
BLS: Bureau of labor statistics
CAS: College alcohol study
MTF: Monitoring the future
NLSY: National longitudinal survey of youth
PRISMA: Preferred reporting items for systematic reviews and meta-analyses
SSRN: Social science research network
UPC: Universal product code
WHO: World Health Organization
