# Supplementary material for: Binge drinking and alcohol prices: a systematic review of age-related results from econometric studies, natural experiments and field studies
Source: Health Econ Rev. 2015 Feb 12;5:6. doi: 10.1186/s13561-014-0040-4 (PMC4384974; doi:10.1186/s13561-014-0040-4)
Supplement: Additional file 1: — Table S1. Youth binge drinking studies (ages < 18 yrs.); Table S2. Young adult binge drinking studies (ages 18–26 yrs.); Table S3. Adult binge drinking studies (ages > 26 yrs.); Table S4. Natural experiments and field interview studies for binge drinking. [file 13561_2014_40_MOESM1_ESM.docx]

**SUPPLEMENTAL TABLES**

**Binge Drinking and Alcohol Prices: A Systematic Review of**

**Age-Related Results from Econometric Studies,**

**Natural Experiments, and Field Studies**

**Supplemental Table S1**. Youth binge drinking studies (ages < 18 yrs.)

| Study | Data,  Ave. age (est.) | Alcohol measure | Price/tax measure | Methods | Controls | Results |
| --- | --- | --- | --- | --- | --- | --- |
| Bhatt (2011) [35] | NLSY, 1997-2003. Age = 16 yrs. | Two or more binge drinking episodes in past 30 days (5+ drinks). | ACCRA beer price at state level, inflation- adjusted. | Probit. Two-stage IV model for drinking & parental transfers. | Demographics, family income, education, **year & region fixed-effects**, etc. | Price is **significant** in four cases. Pseudo R-sqs. are small. |
| Carpenter et al. (2007) [36] | MTF, 1976-2003. Age = 17.5 yrs. | Any binge drinking in past 2 weeks (5+ drinks). | State & federal beer taxes, inflation- adjusted. | Linear probability & logit models. **Robustness tests.** | Demographics, legal age, zero tolerance laws, **year & state fixed-effects.** | Beer tax is insignificant in six cases. |
| Chaloupka & Laixuthai (1997) [37]; Laixuthai & Chaloupka (1993) [44]. **Two studies** | MTF, 1982 & 1989. Age = 17.5 yrs. | Any binge drinking in past 2 weeks (5+ drinks). | State beer tax (1993). ACCRA beer price, adjusted for inflation & city-specific cost-of-living (COL) in 1997 study. | Probit models. Separate results for 1982 & 1989, & pooled sample. | Demographics, income, legal age, border-state age, religion, work status & marijuana laws in 1997 study. | In 1993 study, tax is significant in 1982, but not 1989. In 1997 study, price is significant in 1989, but not in pooled sample. |
| Chatterji (2001) [38] | NLSY, 1997. Age = 15 yrs. | Any binge drinking in past 30 days (5+ drinks). No. of binge episodes in past 30 days. | State beer tax. | Logistic model for participation. OLS model for no. of binge episodes. | Demographics, income, drug use, religion, parents’ education, etc. | Participation, beer tax is insignificant. Frequency, tax is insignificant. |
| Cowan (2011) [39] & private communication with author on 10/29/2013. | NLSY, 1997-2006. Age = 17 yrs. | Number of binge episodes in past 30 days (5+ drinks). | State beer tax, inflation-adjusted. | OLS & IV models for risky behaviors. **Robustness tests**. | Demographics, parents’ education, family income, AFQT score, **year & region fixed-effects.** | Tax coefficient is small & insignificant. |
| Dee (1999b) [40] | MTF, 1977-92. Age = 17.5 yrs. Pseudo-panel data. | Any binge drinking in past 2 weeks (5+ drinks). Prop. in a cell that binge drink. | State & federal beer taxes, inflation- adjusted. | WLS regression model (wts. are respondents per cell). **Tests for robustness**, incl. gender & race effects. | Demographics, legal age, cigarette tax, time trends, **year & state fixed-effects**, etc. | Beer tax is significant without & insignificant or positive with state fixed-effects. |
| Dee (1999c) [41] | MTF, 1977-92. Age = 17.5 yrs. Pseudo-panel data. Sample also for 19 states with beer tax changes. | Any binge drinking in past 2 weeks (5+ drinks). Prop. in a cell that binge drink. | State & federal beer taxes, inflation- adjusted. | WLS regression model (wts are respondents per cell). **Tests for robustness**, incl. tax change model. | Demographics, legal age, **year & state fixed-effects.** Other samples yield similar results. | Beer tax is significant without & insignificant with state fixed-effects. |
| Dee & Evans (2003) [42] | MTF, 1977-92. Age = 17.5 yrs. Pseudo-panel data. | Any binge drinking in past 2 weeks (5+ drinks). Prop. in a cell that binge drink. | State & federal beer taxes, inflation-adjusted. | WLS regression model (wts. are respondents per cell). Two-stage IV model for drinking & education attainment. | Demographics, legal age, **year & state fixed-effects**. **Tests for robustness.** | Tax is negative & significant without fixed-effects & insignificant with state fixed-effects. |
| Grossman (2005) [43] | MTF, 1976-2003. Annual aggregate national data. Age = 17.5 yrs. | Prevalence of binge drinkers in past 2 weeks (5+ drinks). | Bureau of Labor Statistics (BLS) beer price index, inflation-adjusted. | OLS time-series regression. Data not tested for stationarity. | Legal age for beer (population wts.) & time trends. | Beer price is negative & **significant** in two cases. Large elasticities. |
| Markowitz (2001) [45] | National Youth Risk Behavior Survey, 1991, 1993 & 1995. Age = 16 yrs. | Number of binge episodes in past 30 days (5+ drinks). | State beer tax, inflation-adjusted. | Linear probability model. Two-stage model for drinking & violence. | Demographics, income, work status, religion, year effects, etc. **Checks valid instruments.** | Beer tax is negative & **significant** in first-stage results. |
| Medina (2011) [46] | MTF, 1976-2008. Age = 17.5 yrs. Wt. national averages, with subsamples by gender & race. | Prevalence of binge drinkers in past 2 weeks (5+ drinks) in each sample & year. | BLS beer price index, inflation-adjusted. | OLS time-series, linear & double-log regressions. Data not tested for stationarity. | Income, parents’ education, mother’s employment, legal age, risk perceptions, & time trends. | Price of alcohol is generally negative & significant, except for males & non-Whites. |
| Nair et al. (2001) [47] | MTF, 1982 & 1989. Age = 18 yrs. Sub-samples by gender & race. | Any binge drinking in past 2 weeks (5+ drinks). | State & federal beer taxes, adjusted for inflation & city COL. | Probit model by demographic subgroups. | Demographics, income, legal age, religion, mother’s work status, marital status, border-state age, residence, year effects, etc. | Beer tax is significant for males overall, Whites overall, & White males. Insignificant for females overall, Blacks overall, Black males & females. |
| Nelson (2008) [48] | National Survey on Drug Use & Health, 1993-2003, at state level. Age = 15 yrs.; range 12-17 yrs. | Prevalence of binge drinking by state (5+ drinks). State- level adult binge rate (“wetness”) as control. | State beer tax, inflation- adjusted. State laws on alcohol availability & drink-driving. | Linear probability model. Models fit with & without **state fixed-effects**. | Demographics, income, poverty, education, outlet density, etc., availability, adult binge rate, yr fixed-effects. | Tax is negative & significant without fixed effects, but insignificant with state fixed-effects. |
| Renna (2007) [49] | NLSY, 1982-84 for high school seniors. Age = 17.5 yrs. | Two or more binge drinking episodes in past 30 days (6+ drinks). | State beer tax (inflation-adjustment not reported). | Probit model. Two-stage IV model for drinking & on-time graduation. | Demographics, income, legal age, parents’ drinking, AFQT score, **state fixed-effects**. | Beer tax is insignificant. |
| Saffer & Dave (2006) [50]; **two samples** | MTF, 1996 & 1998. Age = 15 yrs. MTF sub-samples by gender & race. NLSY, 1997. Age = 15 yrs. | Any binge drinking in past 30 days (5+ drinks). | ACCRA wt ave. price per ounce of ethanol, adjusted for inflation & city COL. | Probit model for each sample. **Robustness tests** **for NLSY sample**. | Demographics, income, region, education, religion, **year &** **state fixed- effects**, etc. | MTF, price is significant for full sample, Whites only & females only. Insignificant for males & Blacks. NLSY, price is insignificant in four cases. |
| Xuan et al. (2013) [51] | National Youth Risk Behavior Survey, 1999-2009. Age = 16 yrs. | Any binge drinking (5+ drinks). State-level adult binge rate as control. | State beer tax & state sales tax, when applied (no inflation-adjustment reported). | Logistic model, with robust std. errors. Tax rate interactions with adult binge rate. | Demographics, grade level, etc., state-level var. for income, etc. outlet density & adult bingeing. | Beer tax is significant when adult binge rate excluded, but insignificant if it is included. |

**Supplemental Table S2.** Young adult binge drinking studies (ages 18-26 yrs.)

| Study | Data,  Ave. age (est.) | Alcohol measure | Price/tax measure | Methods | Controls | Results |
| --- | --- | --- | --- | --- | --- | --- |
| Bray (2000, 2005) [52,53] | NLSY, 1982-89. Males only, age = 22 yrs. | Binge drinking on 3 or more occasions in past 30 days (6+ drinks). Model with cumulative alcohol use as a control. | State beer tax, inflation-adjusted. | Logit model & discrete factor method (DFM) to control for endogeneity  & sample selection. Two-stage model for drink & wages. | Demographics, religion, legal age, work status, education, cigarette tax, etc. **Tests for robustness**. | Men, tax is negative & significant in logit model. Tax is insignificant in DFM model. |
| Chaloupka & Wechsler (1996) [54] | Harvard College Alcohol Study, 1993. Age = 21 yrs. Subsamples for legal age & underage students. | Any binge drinking in past 2 weeks (5/4+ drinks). | ACCRA beer price for nearest city, adjusted for city cost-of living (COL). | Probit model. Results for full & limited models. Results by age & gender subsamples. | Demographics, Greek status, marital status, religion, region, availability, college-type, drink-driving law index, etc. | Men, price is insignificant for underage & legal age people. Women, price is insignificant for underage & legal age people. |
| Cook & Moore (1994) [55] | NLSY, 1984. Samples for ages 19-22 yrs. & 25-26 yrs. Age = 20.5 yrs. & 25.5 yrs. | Binge drinking on at least 4 occasions in past 30 days (6+ drinks). | State beer tax. | Logistic models for young & older samples. Samples for men, women, 21-22 yrs., & combined. | Demographics, parents’ education, religion, family size, legal age, etc. | Men, tax is insignificant in 5 of 6 regressions. Women, tax is significant for older women. Combined, tax is insignificant in 4 of 5 cases. |
| Cook & Moore (2001) [56] | NLSY, 1982-85 & 1988-89. Age = 18.5 yrs. | Binge drinking on at least 4 occasions in past 30 days (6+ drinks). State drinking variable for per capita ethanol consumption. | State beer tax, inflation-adjusted. | Probit models, short- & long-form results, & gender-specific results. Reports policy model results without & with **state fixed-effects**. | Demographics, income, parents’ education, legal age, religion, marital status, region, work status, parents’ drinking, family size, residence, etc. | Men, tax is insignificant. Women, tax is insignificant. Combined, tax is insignificant. In policy model, tax insignificant with state “wetness” added |
| Cowell (2006) [57] | NLSY, 1982-94. Males only, age = 23 yrs. | Any binge drinking in past 30 days (6+ drinks) & frequent bingeing conditional on binge drink (4+ occasions). | State beer tax, inflation-adjusted. | Probit model. Two-stage DFM model for education & health behavior, with & without unobserved heterogeneity controls. | Demographics, parents’ education, religion, family structure, region, cigarette price, etc. **Robustness tests**. | Men, any binge drinking, tax insignificant in both regressions. Frequent binge drinking, tax is insignificant in both regressions. |
| French & Maclean (2006) [58] | National Epidemiological Survey on Alcohol, 2001-02. Age = 19 yrs. | No. of days respondent felt intoxicated in past year (mean = 10.17 days). | State beer tax. | OLS model, with separate results for men & women. Two-stage IV model for drinking & delinquency. | Demographics, income, education, work status, family size, parents’ drinking, etc. | Men, tax is negative & significant. Women, tax is insignificant. |
| Gius (2003) [59] | NLSY, 1982 & 1994. Control states excluded. Age = 24 yrs. | Any binge drinking in past 30 days (6+ drinks). | State wt. ave. tax per gal. (share wts.), divided by state alcohol price. Bureau of Labor Statistics (BLS) real price of alcohol by region. | Logit model, with state tax rate & regional price both included. | Demographics, income, education, region, family drinking, legal age, etc. | Tax rate & price are insignificant. |
| Grossman et al. (1987) [60] | National Health & Nutrition Examination Survey, 1971-74. Age = 18.5 yrs. | Category variable, no. of drinks on a typical drinking day (1-2, 3-5, 6+ drinks). | BLS prices for beer, wine & spirits for 1971. Extrapolated for 1972-74 & not deflated. | Multinomial logit model. Separate results for each drink category & beverage. | Demographics, income, legal age, & border-state age. | Prices of beer & wine are insignificant for all 3 categories. Price of liquor is insignificant for 6+ drinks. |
| Keng & Huffman (2007) [62]; Keng (1998) [61] | NLSY, 1979-94. Age = 25 yrs. | Binge frequency none (0); moderate (1-3 times); & heavy (4+ times) in past 30 days (6+ drinks). | ACCRA wt. ave. real price per gal. ethanol. ACCRA real price of beer (Keng 1998). | Ordered probit model. Two-stage IV model for drinking & annual labor earnings. | Demographics, work earnings, health status, marital status, religion, legal age, etc. **Year & state fixed- effects.** | Price of alcohol is significant. Short- & long-run elasticities are substantial. In Keng (1998), beer price is insignificant. |
| Kenkel (1993) [63] | Health Promotion & Disease Prevention suppl. to Health Interview Survey, 1985. Sample for ages 18-21 yrs. Ave. age = 19.5 yrs. | No. of binge days in past year (5+ drinks). | ACCRA wt. ave. price for three beverages (budget share wts.), deflated by city COL. | Tobit model. Two-stage model for drinking & drink driving. Results for males & females. | Demographics, income, legal age, education, marital status, health knowledge, drink-driving laws, border-state age, state alcohol laws, etc | Men, price is insignificant. Women, price is significant & elasticity is substantial. |
| Nelson (2008) [48] | National Survey on Drug Use & Health, 1993-2003, at state level. Ave. age = 21.5 yrs.; range 18-25 yrs. | Prevalence of binge drinking by state (5+ drinks). | State beer tax, inflation- adjusted. State laws on alcohol availability & drink-driving. | Linear probability model. Models fit with & without state fixed-effects. | Demographics, income, poverty, education, outlet density, region, availability, etc., **year & state fixed-effects**, | Tax is insignificant or incorrectly signed, with or without state fixed-effects. |
| Powell et al. (2002) [64] | Harvard College Alcohol Study, 1997 & 1999. Undergrads, age = 21 yrs. | Any binge drinking in past 2 weeks (5/4+ drinks). Binge frequency is 3 or more episodes in past 2 weeks. | Two prices at college level: average real price, self-reported; & proportion who pay fixed fee for all they can drink. | Probit models. Two-stage model for drinking & violence. Tests for endogeneity. | Demographics, residence, parents’ education parents drinking, school year, campus type, availability, happy hour restrictions, etc. | **Ave. price is significant for participation & frequency, but small in magnitude.** Fixed fee is positive for any participation & insignificant for frequency. |
| Rhoads (2010) [65] | BRFSS, 1991-2004. Samples for 18-20 yrs. & 21-24 yrs. Ages = 19 yrs. & 22 yrs. | Any binge drinking in past 30 days (5+ drinks). No. of binge episodes in past 30 days (count data). | ACCRA wt. ave. real price per ounce of ethanol. | Probit model for any bingeing. OLS model for frequency, but ignores count data feature | Demographics, income, marital status, work status, region, education, cigarette tax, year fixed-effects, etc. | Any bingeing & binge frequency, alcohol price is insignificant for both age groups. |
| Sutton & Godfrey (1995) [66] | UK, General Household Survey (GHS), 1978-90. Males, age = 21 yrs.; age range is 18-24 yrs. | GHS drinking categories, three highest for units/week are: 22-35, 36-50, & 51+ drinks. The “at-risk” group is > 21 units per week. | National price index: alcohol expenditure at current prices divided by expenditure at 1985 prices, deflated by all items price index. | Grouped data regression for seven categories of drinkers. **Robustness tests** for log model, OLS, etc. Interactions between price & income. | Demographics, income, wealth, education, marital status, work status, peers’ drinking, health status, smoking status, etc. | **Men, price is significantly negative** & interactions are significant, but difficult to interpret. A 5% price increase has a large impact on at-risk group. |
| Wechsler et al. (2000) [67] | Harvard College Alcohol Study, 1997. Underage students < 21 yrs.; age = 20 yrs. | Any binge drinking in past 2 weeks (5/4+ drinks). | Price per drink, self-reported in four categories: > $3, $1-$3, < $1 or set fee; & usually free. | Logistic model, Generalized Estimating Equation (GEE) for robust std. errors. Partial models. Full model not reported. | Demographics, region, & response rate. | **Lower prices or set fee are significant** for probability of binge drinking. Free drinks are significantly likely *to reduce* bingeing. |
| Weitzman et al. (2003) [68] | Harvard College Alcohol Study, 1999. College freshman, aged ≤ 19 yrs. Age = 18.5 yrs. | Any binge drinking in past 2 weeks (5/4+ drinks). | Price variable: “usually pay $1 or less per drink or set fee.” | Logistic regression, with GEE for std. errors. Only final model reported | Demographics, Greek status, peers’ drinking, availability, wet setting, college type, etc. | Final regression, **low price or set fee significantly associated** with binge drinking. |
| Williams et al. (2005) [69] | Harvard College Alcohol Study, 1997 & 1999. Undergrads, < 25 yrs.; age = 21 yrs. | Heavy drinker: > 5/4+ drinks per occasion. Index for no. of drunk events in past 30 days: none; less than 4; & more than 4 events. | Two prices at college level: ave. real price (excl. zeros), self-reported; & prop. paying fixed fee for all they can drink. | Ordered logit models **Robustness tests for campus drinking bans & state fixed-effects.** | Demographics, religion, parents drinking, college type, cigarette price, education, availability, **state fixed- effects**, etc. | **Higher price reduces odds of heavy drinking** & drunk events. Fixed fee is insignificant with state fixed- effects included. |
| Wolaver (2007) [70] | Harvard College Alcohol Study, 1997. Age = 21 yrs. | Any binge drinking in past 2 weeks (5/4+ drinks). Any drunk events in past 30 days. | Three price measures: ave. college price (excl. zeros), self-reported; prop. of students who pay fixed fee; & prop.  who drink free. | Probit model. Two-stage IV model for drinking & college grade pt. ave. Results by gender. | Demographics, religion, Greek status, family income, parents’ drinking, access, cigarette use, college type, etc. | Men, price variables are insignificant for bingeing & drunkenness; free drinks *reduce* bingeing. Women, all price variables are insignificant. |
| Wolaver et al. (2007a) [71]; see also Wolaver et al. (2007b) [72] | Harvard College Alcohol Study, 1997 & 1999. Age = 21 yrs. | Any binge drinking in past 2 weeks (5/4+ drinks). Frequent bingeing is 2+ times or more in past 2 weeks. | Two real prices at college level: ave. real price (excl. zeros), self-reported; & prop. paying fixed fee for all they can drink. | Probit model. Two-stage IV model for bingeing & college-level (peer) binge rate. | Demographics, religion, marital status, Greek status, region, residence, income, parents’ drinking, availability, college type, state & local alcohol policies, etc. | Probit, both prices are insignificant for binge & frequent binge, when college-binge rate is included. First-stage results, price is significant & fixed fee is insignificant. |

ACCRA = American Chamber of Commerce; MTF = Monitoring the Future; NLSY = National Longitudinal Survey of Youth

BRFSS = Behavioral Risk Factor Surveillance System.

**Supplemental Table S3.** Adult binge drinking studies (ages > 26 yrs.)

| Study | Data,  Ave. age (est.) | Alcohol measure | Price/tax measure | Methods | Controls | Results |
| --- | --- | --- | --- | --- | --- | --- |
| Asgeirsdottir et al. (2012) [73] | Iceland Health & Lifestyle Survey, 2007 & 2009. Age = 45 yrs.; age range 18-79 yrs. | Binge drinking at least once a month in past year (5+ drinks). | Price index from Statistics Iceland, inflation-adjusted. | Probit model for change in binge status, net of time-varying covariates. Also SUR & linear prob. models. | Demographics,  income, hrs. worked, health status, marital status, residence, education, etc. | Binge elasticity is negative, but not significant. |
| Ayyagari et al. (2013) [29] | Health & Retirement Study, 1996-2004. Age = 65 yrs.; age range 51 – 81+ yrs. | Ave. number of drinks per day & number of binge days in past 3 months (4+ drinks). | ACCRA ave. price at state level, adjusted for ethanol content per drink, state cost-of-living (COL) & inflation. | Two- & three-component finite mixture model (FMM), with Poisson-distributed subpopulations. Two latent groups found. | Demographics, income, risk aversion, health, education, work status, marital status, etc. **Robustness tests** include state laws on drink driving, beverage prices, etc. | Moderate drinkers are price responsive, & more likely to be older, non-white, female, married & less educated. Heavier drinkers are insensitive to price & more likely to binge. |
| Byrnes et al. (2013) [75] & Web appendix | Australia, National Drug Strategy Household Survey, 2001, 2004 & 2007. Age = 45 yrs. | Number of days alcohol was consumed at four intensities for std. drinks per occasion (0, 1-4, 5-9, 10+ drinks). | Price index for alcohol products from national sales data, adjusted for state consumer price index (CPI) & inflation. | Pooled 3SLS for each of four intensities. Coefficients constrained to sum to zero. | Demographics, income, work status, marital status, year education, residence, etc. | Frequency of consumption is responsive to price at lowest intensities, but insignificant at two highest (5-9, 10+ drinks). |
| Cook (2007, pp. 73-75) [76] | NESARC, 2001-2002. Age range = 18 yrs. & older. Control states excluded. | Any binge drinking in past year (5/4+ drinks). State  drinking measured by ave. per capita ethanol sales. | State-level tax index for 2000, based on tax rate & ethanol content for each beverage. | Logit model for men & women separately. Reports percent change in odds of bingeing. Subsamples estimated, but not reported. | Demographics, income, work status, marital status, health status, education, height, weight, & state “wetness.” | **Tax coefficients are negative & significant for both men & women**. Effects are substantial for both genders. |
| Davalos et al. (2012) [77] | NESARC, 2001-02 & 2004-05. Age = 46 & 49 yrs. | Any binge drinking in past year. No. of binge days in past year (5/4+ drinks). Dependent variables also for drink-driving & alcohol dependence. | State beer tax per 12-oz. drink, inflation-adjusted. | Conditional logit model for participation & conditional negative binomial model for frequency, with controls for **state & individual fixed-effects.** | Demographics, income, marital status, health status, state unemployment, residence, state ethanol per capita, etc. **Robustness tests** for state unemployment & month of interview. | Tax is negative & significant for any bingeing in one of two regressions. **Tax is negative & significant for binge freq.** Beer tax is insignificant for two other drink measures. |
| Dee (1999a) [78] | BRFSS, 1984-95. Age = 45.5 yrs.; range 18 to 56+ yrs. Pseudo-panel data. Sub-samples by age, gender, race & work status. | Any binge drinking in past 30 days (5+ drinks). | Real state & fed. beer tax; state & fed. liquor tax for license states; & wt. ave. tax per gal. ethanol for license states. | Linear probability model for pseudo-panel data, with & without state fixed-effects for full sample. | Demographics, family income, parents’ education, marital status, state income, state trends & **year & state-fixed effects**. | All taxes are insignificant or incorrectly positive in 8 of 9 cases for full sample. Taxes are insignificant for men & women samples. |
| Gius (2002) [79] | National Longitudinal Survey of Youth, 1994. Age = 31 yrs.; range 29-33 yrs. | Any binge drinking in past 30 days (6+ drinks). Total & drinkers only samples. | State-level alcohol taxes for each beverage in dollars per gal. Excludes control states. | Probit model, with all three taxes included. | Demographics, income, marital status, debt load, education, work status, etc. | All three taxes are insignificant in four regressions for binge drinking. |
| Kenkel (1993) [63] | Health Promotion & Disease Prevention suppl. to 1985 Health Interview Survey. Age = 42.5 yrs. for men & 44 yrs. for women. | No. of binge days in past year (5+ drinks). | ACCRA wt. ave. price for three beverages (budget share wts.), deflated by city COL. | Tobit model. Two-stage model for drinking & drink driving. Results for males & females. | Demographics, income, education, health status & knowledge, marital status, state drink-driving & alcohol laws, etc | **Men, price is negative & significant**. **Women, price is negative & significant**. Substantial elasticities. |
| Kenkel (1996) [80] | Health Promotion & Disease Prevention suppl. to 1985 Health Interview Survey. Age = 43 yrs. for men & 46 yrs. for women. | No. of binge days in past year (5+ drinks). | ACCRA wt. ave. price for three beverages (budget share wts.), deflated by city COL. | Tobit model, with separate results for males & females. **Robustness test** with two-stage model for non-zero demands. | Demographics, income, marital status, border price, state drink driving & alcohol laws, etc. Health knowledge about heavy drinking, interacted with price. | Direct price effect is insignificant for men & women, but interaction with knowledge is significant. Price effects are significant for drinkers with average or better knowledge, but insignificant for poor-informed. |
| Ludbrook et al. (2012) [81] | United Kingdom, Expenditure & Food Survey, 2006-08. Age = 51 yrs. | Harmful drinking is 50+ units per wk. for men & 35+ units per wk. for women per UK Guidelines. | Estimates of no. units of alcohol purchased & no. purchased at under £0.45 ($0.89USD), off-trade. | Two-part model: probit model for off-trade buys; & tobit model for number of cheap units purchased, off-trade | Age, income, no. of children, & survey year. Pseudo R-sq. is very small. | Relative to moderate drinkers, **harmful drinkers purchased significantly more units** of cheap alcohol. |
| Manning et al. (1995) [31]; see also Blumberg (1992) | Alcohol & Health Practices suppl. to 1983 Health Interview Survey. Age = 39.5 yrs. | Any binge drinking in past year. Number of binge days in past year (5+ drinks). | ACCRA wt. ave. real price per unit of ethanol, adjusted for sales tax & ave. of the Bureau of Labor Statistics & ACCRA COL. | Two-part model: logit model for being a binge drinker & conditional OLS for frequency of binge drinking. Some results for quantile model. | Demographics, income, family size, education, residence, region, etc. Price response in quantile model diminishes as drinks increase.. | Price elasticity for participation is significant, but conditional & total elasticities not. Price response for no. of ounces is insignificant. |
| McLellan (2011) [82]; see also McLellan et al. (2012) | BRFSS, 2001-05. Age = 45 yrs. Sub-samples by gender, but beer price results not reported. | Any binge drinking in past 30 days (5+ drinks). | ACCRA beer price, unwt. state average, inflation-adjusted. | Logit model for total sample & linear prob. model for gender results. **Robustness tests for sample wts.** | Demographics, poverty status, partner status, work status, education, cigarette price, etc., **regional & state fixed-effects**. | Price is significantly less than one with regional fixed-effects, but insignificant with state fixed-effects included. |
| Nelson (2008) [48] | National Survey on Drug Use & Health, 1993-2003, at state level. Age = 45 yrs. | Prevalence of binge drinking by state (5+ drinks). | State beer tax, inflation- adjusted. State laws on alcohol availability & drink-driving. | Linear probability model. Models fit with & without **state fixed-effects.** | Demographics, income, poverty, education, outlet density, region, availability, etc., | Tax is insignificant in models with or without state fixed-effects. |
| Popovici & French (2013) [83]; & private communication with authors on 12/04/2013 | NESARC, 2001-02 & 2004-05. Age = 44 yrs.; age range = 21 -65 yrs. Excludes pregnant women & homemakers. | Number of binge drinking days in past year (5/4+ drinks). Also diagnosis for alcohol dependence. | ACCRA prices for beer, wine & spirits (each), adjusted for state COL index. | Negative binomial model for pooled panel data. Separate gender results. | Demographics, income, marital & work status, education, smoking, drug use, etc. & **state fixed-effects**. | IRRs for prices are insignificant. Men & Women samples, prices are insignificant or positive. |
| Rhoads (2010) [65] | BRFSS, 1991-2004. Age = 45 yrs. Subsamples for ages 25-39, 40-64 & 65+ yrs. | Any binge drinking in past 30 days (5+ drinks). No. of binge days in past 30 days (count data). | ACCRA wt  ave. real price per ounce of ethanol, | Probit model for any binge participation. OLS model for frequency, but ignores count data feature. | Demographics, income, marital status, work status, education, cigarette tax, year fixed-effects**,** etc. | Participation, alcohol price is insignificant or positive. Frequency, price is negative & significant for 40-64 & 65+ yrs |
| Sloan et al. (1995) [84] | BRFSS, 1984-90. Age = 34 yrs. | Any binge drinking in past 30 days. No. of binge days in past 30 days (5+ drinks). | ACCRA wt. ave. real price of alcohol at state level, expressed relative to California in 1990. | Two-part model: probit model for participation; Tobit or OLS models for frequency. | Demographics, education, legal age, income & 12 variables for drink-driving laws & enforcement. | Participation, alcohol price is insignificant. Frequency, price is negative & significant in tobit, but not in OLS model. |
| Stout et al. (2000) [85] | BRFSS, 1984-95. 25% random sample for ages > 21 yrs. Age = 42.5 yrs. | Any binge drinking in past 30 days (5+ drinks). | ACCRA wt  ave. real price of alcohol at state level, expressed relative to California in 1990. | Logit model for binge drinking & binge drinking & driving. | Demographics, income, education, health behavior, state features, outlet density, time trends & 17 variables for drink-drive laws | Alcohol price is insignificant for probability of binge drinking, given person is a drinker. |
| Terza (2002) [86] | Alcohol & Health Practices suppl. to 1988 Health Interview Survey. Males, age = 40 yrs. | Individual exceeds sample 90% percentile of ethanol use in past 2 weeks. State” wetness” is per capita ethanol sales. | State beer tax. | Probit model. Two-part model for alcohol abuse (probit) & work status (MNL logit). | Demographics, health status, education, work status, parents’ drinking, region, state “wetness,” cigarette tax, etc. | Men, beer tax & its square are statistically insignificant. |
| Zhang (2010) [87] | BRFSS, 1985-2002. Females, age = 30 yrs. (est.); age range < 44 yrs. | Any binge drinking by a pregnant woman in past 30 days (5+ drinks). Incidence is only 1.4%. | Federal & state real alcohol taxes for beer, wine & spirits. Excludes control states for spirits. | Two-part model: probit & OLS. Separate results reported for each tax rate by beverage. | Demographics, income, marital status, education, **year & state fixed-effects.** | **Women, all three tax rates are negative & significant**, with sizeable elasticities. |

NESARC = National Epidemiological Survey on Alcohol & Related Conditions

**Supplemental Table S4.** Natural experiments and field interview studies for binge drinking

| Study | Data,  Ave age (est.) | Alcohol measure | Price/tax measure | Methods | Controls | Results |
| --- | --- | --- | --- | --- | --- | --- |
| Chung et al. (2013) [88] | Hong Kong, special telephone surveys in 2006, 2011 & 2012. Age = 44 yrs. | Any binge drinking in past 30 days (5/4+ drinks). Also alcohol abuse & dependence variable. | Natural experiment, govt. excise tax cut of 50% in 2007 & 100% cut in 2008 on beer & wine. | Logistic model, with year effect for 2011 & 2012 compared to 2006 (odds greater than one is positive effect of cuts). | Demographics, marital status, work status, education & year. Binge reductions not due to global economic crisis. | Odds ratio less than one for 2011; insignificant for 2012. Prevalence fell from 2006 to 2011-12. |
| Clapp et al. (2003) [89] | US, two telephone surveys of college students, 2000-01. Ages = 24 yrs. & 20 yrs. | Any binge drinking in the past 28 days (5+ drinks). Subjective rating for “felt drunk.” | Free (no-cost) alcohol provided at event (private parties, bar, & public gatherings). | Discriminant factor analysis & path-analysis model. Exploratory- confirmatory analyses. | Environmental & personal factors for food, risk, bartender, drugs, BYOB, games, gender, etc. | Free alcohol not important for factor loadings & does not appear in path-analysis models. |
| Gmel et al. (2008) [90] | Switzerland, 1999- 02, four-wave telephone survey of 889 alcohol users, one pre- and 3 post-tax surveys. Age = 15-60+ yrs. | Drinkers divided into three groups by ethanol per day: high consumers are males > 40g & female > 20g. | Natural experiment, tax reduction of 9-50% on foreign spirits & 30-50% reduction on price of foreign spirits | Change in consumption of spirits & total alcohol. Three controls for regression to mean (RTM) & hierarchical linear model. | Demographics. Only those who remained drinkers are retained in sample. Time-points matter for drinking categories. | Adjusted for RTM, high-level drinkers increased consumption in the short term, but effect was not lasting. |
| Gustafsson (2010) [91]; see also Makela et al. (2007) | Sweden, telephone survey during 2003, 2004, 2005& 2006. Age ranges: 16-49 yrs. & 50-80 yrs. | Binge drink is one or more bottles of wine, 5 drums of spirits, or 4-6 cans of beer. Identifies also top 10% of heavy drinkers. | Natural experiment, 45% decline in Danish tax on spirits (2003) & Swedish increase in travelers’ allowance. 25% price reduction. | One yr. (03-04), two yr. (03-05) & three yr. (03-06) changes in ave. monthly consumption for binge drinkers & heavy drinkers. | Separate results for men & women, age, social class, south vs. north Sweden (control area). | Insignificant increase in ave. volume for bingers in S. Sweden. Insignificant increases for heavy drinkers, men vs. women & younger drinkers. |
| Heeb et al. (2003) [92]; see also Kuo et al. (2003) [95] | Switzerland, 1999, telephone survey, 3 mths. before & 3 mths. after tax-change. Age = 15-29, 30-59 & 60+ yrs. | Any binge drinking in past 6 months (6/4+ drinks). Restricted to current drinkers. | Natural experiment, tax reduction of 9-50% on foreign spirits & 30-50%  for price reduction on foreign spirits | OLS regressions, with bingers classified into stable drinker, increasers & decreasers. | Any change in binge drinking on before/after basis for three age groups & males & females. | Some increase among men. Any changes were not significant controlling for age, sex, and volume. |
| Helakorpi et al. (2010) [93]; see also Mustonen et al. (2007) | Finland, Health Behaviour & Health Surveys, 1982-2008. Age = 45 yrs.; range 25-64 yrs. | Binge drinking at least once a week for men & once a month for women (6+ drinks). | Natural experiment, reduction by 33% in excise taxes in 2004 & increase in travelers’ allowance. | Logistic model: age-adjusted odds ratios for 2003-08 & 2004-08. | Age, gender, & education attainment. Drinking increased more among 45-64 age group. | Men, binging increases for lowest group by education only. . Women, increases for highest group by education only. |
| Jamison & Myers (2008) [94] | UK, survey of UCL students, 2006. Age = 23 yrs. | Any binge drinking in a typical week (5/4+ drinks). | Prices are measured by “buying rounds” & anticipated behavior for “special offers.” | Step-wise OLS. Mann-Whitney (M-W) tests for indep. groups for binge & non-binge drinkers. | Demographics, peer influence, intentions, attitudes, norms, school status, etc. | Prices do not appear in regressions. M-W test indicate bingers respond to special offers. |
| O’Mara et al. (2009) [96] | US, 2008, random & self-selected students, bar district near large college campus, 4 nights. Age = 22 yrs. (est.). | Breath test (BrAC) to assess intoxication. Self-report on grams ethanol consumed & expenditures. | Mean est. cost per gram falls from $0.23 for the BrAC < 0.08g group to $0.16 for BrAC ≥ 0.08g group. | Logistic model, with fixed- and random-effects. Dep. variable is intoxication. | Gender, body mass, no. of drinks consumed on-& off-premise, intentions, etc. | **On-premise cost is significant**; & off-premise cost is not significant for intoxication. |
| Stockwell et al. (1993) [97] | Australia, 1990, special household survey of drinking settings. Age =25 yrs (est.). | Any binge drinking in past 3 months (6/4+ drinks). | Price- discounting on drinks. | Step-wise logistic model for 321 drinkers with heaviest consumption and/or harm outcome. | Demographics, marital status, location, & drinking setting, etc. | Price- discounting is insignificant for binge drinking & alcohol-harms. |
| Thombs et al. (2008, 2009)  [98,99] | US, 2007, random & self-selected students, bar districts near two large colleges for 4 nights. Age = 21 yrs. | Breath test (BrAC) to assess intoxication. | Three price measures: all-you-can drink at fixed price; drink game discounts; & reduced prices on specials. | Multivariate model, with random-effects for each bar. | Demographics, night of week, sampling status, etc. All-you-can-drink includes zeros for females. | **Fixed price statistically significant**. Other price promotions are not significant. |
| Wagoner et al. (2012) [100] | US, 2003-2005, annual Web-based survey of students at 10 colleges. Age = 20 yrs. | Any binge drinking in past 30 days (5/4+ drinks). | Variable for frequency of free drinks: never; seldom; & often. | Logistic model. Results for gender interactions with price. | Demographics, yr. in school, Greek status, relationship status, monthly allowance, etc. | **Free drinks are positive- significantly associated** with odds of bingeing for both genders. |

BYOB = bring your own booze
