# Supplementary material for: Binge drinking and alcohol prices: a systematic review of age-related results from econometric studies, natural experiments and field studies
Source: Health Econ Rev. 2015 Feb 12;5:6. doi: 10.1186/s13561-014-0040-4 (PMC4384974; doi:10.1186/s13561-014-0040-4)
Supplement: Additional file 2: — Primary study references. [file 13561_2014_40_MOESM2_ESM.docx]

**Additional file 2: Primary Study References**

(** indicates supporting/duplicate articles not reviewed in Table 2)

Asgeirsdottir TL, Corman H, Noonan K, Olafsdottir P, Reichman NE (2012) Are recessions good for your health behaviors? Impacts of the economic crisis in Iceland. NBER Working Paper 18233. National Bureau of Economic Research, New York.

Ayyagari P, Deb P, Fletcher J, Gallo W, Sindelar JL (2013) Understanding heterogeneity in price elasticities in the demand for alcohol for older individuals. Health Econ 22: 89-105.

Bhatt V (2011) Adolescent alcohol use and intergenerational transfers: evidence from micro data. J Family Econ Issues 32: 296-307.

**Blumberg LJ (1992) Second best alcohol taxation: balancing appropriate incentives with deadweight loss. Unpublished PhD dissertation. University of Michigan, Ann Arbor.

Bray JW (2000) Identifying inputs to the human capital production function: the effects of alcohol use on human capital formation. Unpublished Ph.D. dissertation. University of North Carolina, Chapel Hill.

Bray JW (2005) Alcohol use, human capital, and wages. J Labor Econ 23: 279–312.

Byrnes J, Shakeshaft A, Petrie D, Doran C (2013) Can harms associated with high-intensity drinking be reduced by increasing the price of alcohol? Drug Alcohol Rev 32: 27-30.

Carpenter CS, Kloska DD, O’Malley P, Johnston L (2007) Alcohol control policies and youth alcohol consumption: evidence from 28 years of Monitoring the Future. BE J Econ Analysis Policy 7: Art. 25.

Chaloupka FJ, Laixuthai A (1997) Do youths substitute alcohol and marijuana? Some econometric evidence. East Econ J 23: 253-276.

Chaloupka FJ, Wechsler H (1996) Binge drinking in college: the impact of price, availability, and alcohol control policies. Contemp Econ Policy 16: 112-124.

Chatterji P (2001) What determines adolescent demand for alcohol and marijuana? A comparison of findings from the NLSY79 and the NLSY97, in *Social Awakening: Adolescent Behavior as Adulthood Approaches* (Michael RT ed.), pp. 299-338. Russell Sage Foundation, New York.

Chung VCH, Yip BHK, Griffiths SM, Yu ELM, Kim JH, Tam WWS, Wong AHC, Chan IWT, Lau JTF (2013) The impact of cutting alcohol duties on drinking patterns in Hong Kong. Alcohol Alcohol (in press). doi: 10.1093/alcalc/agt065.

Clapp JD, Lange J, Min JW, Shillington A, Johnson M, Voas R (2003) Two studies examining environmental predictors of heavy drinking by college students. Prev Sci 4: 99-108.

Cook PJ (2007) Paying the Tab: The Costs and Benefits of Alcohol Control. Princeton University Press, Princeton.

Cook PJ, Moore MJ (1994) This tax’s for you: the case for higher beer taxes. National Tax J 47: 559-573.

Cook PJ, Moore MJ (2001) Environment and persistence in youthful drinking patterns, in *Risky Behavior Among Youth: An Economic Analysis* (Gruber J ed.), pp. 375-427. University of Chicago Press, Chicago.

Cowan BW (2011) Forward-thinking teens: the effects of college costs on adolescent risky behavior. Econ Educ Rev 30: 813-825.

Cowell AJ (2006) The relationship between education and health behavior: some empirical evidence. Health Econ 15: 125-146.

Davalos ME, Fang H, French MT (2012) Easing the pain of an economic downturn: macroeconomic conditions and excessive alcohol consumption. Health Econ 21: 1318-1335.

Dee TS (1999a) Taxes, alcohol use and traffic fatalities. Unpublished working paper. Swarthmore College, Swarthmore.

Dee TS (1999b) The complementarity of teen smoking and drinking. J Health Econ 18: 769-793.

Dee TS (1999c) State alcohol policies, teen drinking and traffic fatalities. J Pub Econ 72: 280-315.

Dee TS, Evans WN (2003) Teen drinking and educational attainment: evidence from two-sample instrumental variables estimates. J Labor Econ 21: 178-209.

French MT, Maclean JC (2006) Underage alcohol use, delinquency, and criminal activity. Health Econ 15: 1261-1281.

Gius MP (2002) The effect of taxes on alcohol consumption: an individual level analysis with a correction for aggregate public policy variables. Penn Econ Rev 11: 76-93.

Gius MP (2003) Using NLSY-Geocode data to determine the effects of taxes and minimum age laws on the alcoholic beverage demands of young adults. New York Econ Rev 34: 38-50.

Gmel G, Wicki M, Rehm J, Heeb J-L (2008) Estimating regression to the mean and true effects of an intervention in a four-wave panel study*.* Addiction 103: 32-41.

Grossman M (2005) Individual behaviours and substance use: the role of price, in *Substance Use: Individual Behaviour, Social Interactions, Markets and Politics* (Lindgren B, Grossman M eds.), pp. 15-39. Elsevier, Amsterdam.

Grossman M, Coate D, Arluck GM (1987) Price sensitivity of alcoholic beverages in the United States: youth alcohol consumption, in *Control Issues in Alcohol Abuse Prevention: Strategies for States and Communities* (Holder HD ed.), pp. 169-212). JAI Press, Greenwich, CT.

Gustafsson NKJ (2010) Alcohol consumption in southern Sweden after major decreases in Danish spirits taxes and increases in Swedish travellers’ quotas. Euro Addiction Res 16: 152-161.

Heeb J-L, Gmel G, Zurbrugg C, Kuo M, Rehm J (2003) Changes in alcohol consumption following a reduction in the price of spirits: a natural experiment in Switzerland. Addiction 98: 1433-1446.

Helakorpi S, Makela P, Uutela A (2010) Alcohol consumption before and after a significant reduction of alcohol prices in 2004 in Finland: were the effects different across population subgroups. Alcohol Alcohol 45: 286-292.

Jamison J, Myers LB (2008) Peer-group and price influence students drinking along with planned behaviour. Alcohol Alcohol 43: 492-497.

Keng S-H (1998) The demand for health, alcohol abuse, and labor market outcomes: a longitudinal study. Unpublished Ph.D. dissertation. Iowa State University, Ames.

Keng S-H, Huffman WE (2007) Binge drinking and labor market success: a longitudinal study on young people. J Population Econ 20: 35-54.

Kenkel DS (1993) Drinking, driving, and deterrence: the effectiveness and social costs of alternative policies. J Law Econ 36: 877-913.

Kenkel DS (1996) New estimates of the optimal tax on alcohol. Econ Inquiry 34: 296-319.

**Kuo M, Heeb J-L, Gmel G., Rehm J (2003) Does price matter? The effect of decreased price on spirits consumption in Switzerland. Alcohol Clinic Exp Res 27: 720-725.

Laixuthai A, Chaloupka FJ (1993) Youth alcohol use and public policy. Contemp Policy Issues 11: 70-81.

Ludbrook A, Petrie D, McKenzie L, Farrar S (2012) Tackling alcohol misuse: purchasing patterns affected by minimum pricing of alcohol. Applied Health Econ Health Policy 10: 51-63.

**Makela P, Bloomfield K, Gustafsson N-K, Huhtanen P, Room R (2007) Changes in volume of drinking after change in alcohol taxes and travellers’ allowances: results from a panel study. Addiction 103: 181-191.

Manning WG, Blumberg L, Moulton LH (1995) The demand for alcohol: the differential response to price. J Health Econ 14: 123-148.

Markowitz S (2001) The role of alcohol and drug consumption in determining physical fights and weapon carrying by teenagers. East Econ J 27: 409-432.

McLellan DL (2011) Intended and unintended consequences: effects of state cigarette price on smoking and current, binge, and heavy drinking by demographic group. Unpublished Ph.D. dissertation. Brandeis University, Boston.

**McLellan DL, Hodgkin D, Fagan P, Reif S, Horgan CM (2012) Unintended consequences of cigarette price changes for alcohol drinking behaviors across age groups: evidence from pooled cross sections. Sub Abuse Treatment Prev Policy 7: 28. doi: 10.1186/1747-597X-28.

Medina J (2011) Smoking, drinking, and binge drinking: an empirical study of the role of price on consumption by high school seniors. Unpublished Ph.D. dissertation. City University of New York, New York.

**Mustonen H, Makela P, Huhtanen P (2007) People are buying and importing more alcohol than ever before: where is it going? Drug Educ Prev Policy 14: 513-527.

Nair R, Chaloupka FJ, Grossman M, Saffer H (2001) Gender and race differences in youth alcohol demand, in *Economic Analysis of Substance Use and Abuse: The Experience of Developed Countries and Lessons for Developing Countries* (Grossman M, Hsieh C-R eds.), pp. 391-411. Edward Elgar, Cheltenham, United Kingdom

Nelson JP (2008) How similar are youth and adult alcohol behaviors? Panel results for excise taxes and outlet density. Atlantic Econ J 36: 89-104.

O’Mara RJ, Thombs DL, Wagenaar AC, Rossheim ME, Merves ML, Hou W, Dodd VJ, Pokorny SB, Weiler RM, Goldberger BA (2009) Alcohol price and intoxication in college bars. Alcohol Clinic Exp Res 33: 1973-1980.

Popovici I, French MT (2013) Does unemployment lead to greater alcohol consumption? Industrial Relations 52: 444-466.

Powell LM, Czart-Ciecierski CU, Chaloupka FJ, Wechsler H (2002) Binge drinking and violence among college students: sensitivity to correlation in the unobservables. ImpacTeen Research Paper Series No. 20. University of Illinois at Chicago, Chicago.

Renna F (2007) The economic cost of teen drinking: late graduation and lowered earnings*.* Health Econ 16: 407-419.

Rhoads JK (2010) Consequences of tobacco control policies: intended and unintended. Unpublished Ph.D. dissertation. University of Illinois at Chicago, Chicago.

Saffer H, Dave D (2006) Alcohol advertising and alcohol consumption by adolescents. Health Econ 15: 617-637.

Sloan FA, Reilly BA, Schenzler C (1995) Effects of tort liability and insurance on heavy drinking and drinking and driving*.* J Law Econ 38: 49-77.

Stockwell T, Lang E, Rydon P (1993) High risk drinking settings: the association of serving and promotional practices with harmful drinking. Addiction 88: 1519-1526.

Stout EM, Sloan FA, Liang L, Davies HH (2000) Reducing harmful alcohol-related behaviors: effective regulatory methods. J Stud Alcohol 61: 402-412.

Sutton M, Godfrey C (1995) A grouped data regression approach to estimating economic and social influences on individual drinking behaviour. Health Econ 4: 237-247.

Terza JV (2002) Alcohol abuse and employment: a second look. J Applied Econometrics 17: 393-404.

Thombs DL, Dodd V, Pokorny SB, Omli MR, O’Mara R, Webb MC, Lacaci DM, Werch C (2008) Drink specials and the intoxication levels of patrons exiting college bars. Amer J Health Beh 32: 411-419.

Thombs DL, O’Mara R, Dodd VJ, Hou W, Merves ML, Weiler RM, Pokorny SB, Goldberger BA, Reingle J, Werch CE (2009) A field study of bar-sponsored drink specials and their associations with patron intoxication. J Stud Alcohol Drugs 70: 206-214.

Wagoner KG, Blocker J, McCoy TP, Sutfin EL, Champion H, Wolfson M (2012) Free alcohol use and consequences: gender differences among undergraduates. Amer J Prev Med 36: 446-458.

Wechsler H, Kuo M, Lee H, Dowdall GW (2000) Environmental correlates of underage alcohol use and related problems of college students. Amer J Prev Med 19: 24-29.

Weitzman ER, Nelson TF, Wechsler H (2003) Taking up binge drinking in college: the influences of person, social group, and environment*.* J Adolescent Health 32: 26-35.

Williams J, Chaloupka FJ, Wechsler H (2005) Are there differential effects of price and policy on college students’ drinking intensity? Contemp Econ Policy 23: 78-90.

Wolaver AM (2007) Does drinking affect grades more for women? Gender differences in the effects of heavy episodic drinking in college. American Economist 51: 72-88.

Wolaver A, Ciecierski C, Powell L, Wechsler H (2007a) Peer effects and their role in binge drinking across American college campuses. ImpacTeen Research Paper 33. University of Illinois at Chicago.

**Wolaver A, Ciecierski C, Powell L (2007b) What matters: reality or perception? The impact of peer binging on college student drinking behaviors. ImpacTeen Research Paper 36. University of Illinois at Chicago, Chicago.

Xuan Z, Nelson TF, Heeren T, Blanchette J, Nelson DE, Gruenewald P, Naimi TS (2013) Tax policy, adult binge drinking, and youth alcohol consumption in the United States. Alcohol Clinic Exp Res (in press). doi: 10.1111/acer.12152.

Zhang N (2010) Alcohol taxes and birth outcomes. Int J Env Res Pub Health 7: 1901-1912. doi: 10.3390/ijerph7051901.
